# Self-assessment, and not continuous training, improves basic open suturing skills

**DOI:** 10.1080/10872981.2024.2374101

**Published:** 2024-07-01

**Authors:** Vera Hillemans, Otmar Buyne, Ivo de Blaauw, Sanne M.B.I. Botden, Bas H. Verhoeven, Maja Joosten

**Affiliations:** a Department of Surgery, Radboudumc, Nijmegen, The Netherlands; b Department of Pediatric Surgery, Radboudumc – Amalia Children’s hospital, Nijmegen, The Netherlands; c Radboudumc – Amalia Children’s hospital, Nijmegen, The Netherlands

**Keywords:** Basic open suturing tasks, tracking, continuous training, self-assessment, reflection before practice

## Abstract

**Background:**

To develop and maintain suturing skills, clinical exposure is important. When clinical exposure cannot be guaranteed, an adequate training schedule for suturing skills is required. This study evaluates the effect of continuous training, ‘reflection before practice’ and self-assessment on basic open suturing skills.

**Methods:**

Medical students performed four basic suturing tasks on a simulation set up before (‘pre-test’) and after their surgical rotation (‘after-test’). Participants were divided in three groups; the ‘clinical exposure group’ (*n* = 44) had clinical exposure during their rotation only, the ‘continuous training group’ (*n* = 16) completed a suturing interval training during their rotation and the ‘self-assessment group’ (*n* = 16) also completed a suturing interval training, but with the use of reflection before practice and self-assessment. Parameters measured by a tracking system during the suturing tasks and a calculated ‘composite score’ were compared between groups and test-moments.

**Results:**

A significantly better composite score was found at the after-test compared to the pre-test for all groups for all basic suturing tasks (0.001 ≤ *p* ≤ 0.049). The self-assessment group scored better at the pre-test than the other two groups for all tasks, except for ‘knot tying by hand’ (0.004 ≤ *p* ≤ 0.063). However, this group did not score better at the after-test for all tasks, compared to the other two groups. This resulted in a smaller delta of time (‘transcutaneous suture’, *p* = 0.013), distance (‘Donati suture’ and ‘intracutaneous suture’, 0.005 ≤ *p* ≤ 0.009) or composite score (all tasks, except for knot tying by hand, 0.007 ≤ *p* ≤ 0.061) in the self-assessment group.

**Conclusion:**

Reflection before practice and self-assessment during continuous training of basic open suturing tasks, may improve surgical skills at the start of the learning curve.

## Introduction

Basic suturing skills are essential skills to acquire for medical students at medical school. To further develop these skills, clinical exposure during their surgical rotation is required. Clinical exposure to open surgical skills is diminishing [1–3] and hence the acquisition of suturing skills during surgical rotation cannot always be guaranteed. Therefore, it is important to provide adequate surgical training to acquire and maintain surgical skills [4–7].

There is no consensus yet about which training method leads to the best retention of skills; massed training at the start of a surgical rotation or interval training during a surgical rotation. Massed training is defined as when participants receive all instructions and practical training at one training moment, whereas interval training is defined as instructions and practical training that are divided in more training moments. Some studies described no differences in time or number of errors when comparing interval and massed training [8,9], while other studies suggested that interval training resulted in a better improvement than massed training [10–13].

Another method that might improve surgical skills is self-assessment. The influence of self-assessment on learning curves has been studied extensively [14–19]. Most studies investigated the correlation between self-assessment and expert-assessment or the accuracy of a participant’s self-assessment [14–16]. Surgical residents, for example, seem to underestimate their own surgical skills using self-assessment [20]. A few studies focused on the effect of self-assessment on a participant’s learning curve [17–19]. These studies suggested that self-assessment could have a positive influence on skill acquisition.

Self-assessment provides the opportunity of self-reflection. Many studies focus on the effect of self-reflection during and after open surgical skills [21–23]. A few studies investigated the effect of self-reflection before actionfor example, in laparoscopic surgery, dental education and nursing clinical practice [24–26]. However, self-reflection before performing open surgical skills has not been investigated yet. ‘Reflection before practice’ is a systematic approach of self-assessment [24]. By studying a self-assessment form before executing a task, the most important components of the task can be identified. Due to reflection before practice, a trainee could focus on these most important components during execution of the task, resulting in more effective action and decision-making [24].

Due to diminishing clinical exposure of open surgical skills [1–3], it is important to identify an adequate training schedule for open surgical skills. The aim of this study is to evaluate the effect of continuous training with or without self-assessment and the effect of reflection before practice on basic open suturing skills of medical students during their surgical rotation.

## Methods

### Participants

Medical students at the Radboudumc, Nijmegen, the Netherlands, were recruited for this study. All students were included before their eight-week surgical rotation in the period from September 2020 until June 2021. Prior to their surgical rotation and inclusion in the study, they all completed two five-hour basic suturing courses as part of their curriculum. Medical students with suturing experience, defined as more than five sutures performed prior to this rotation and study, were excluded.

### Equipment

**Figure 1.**
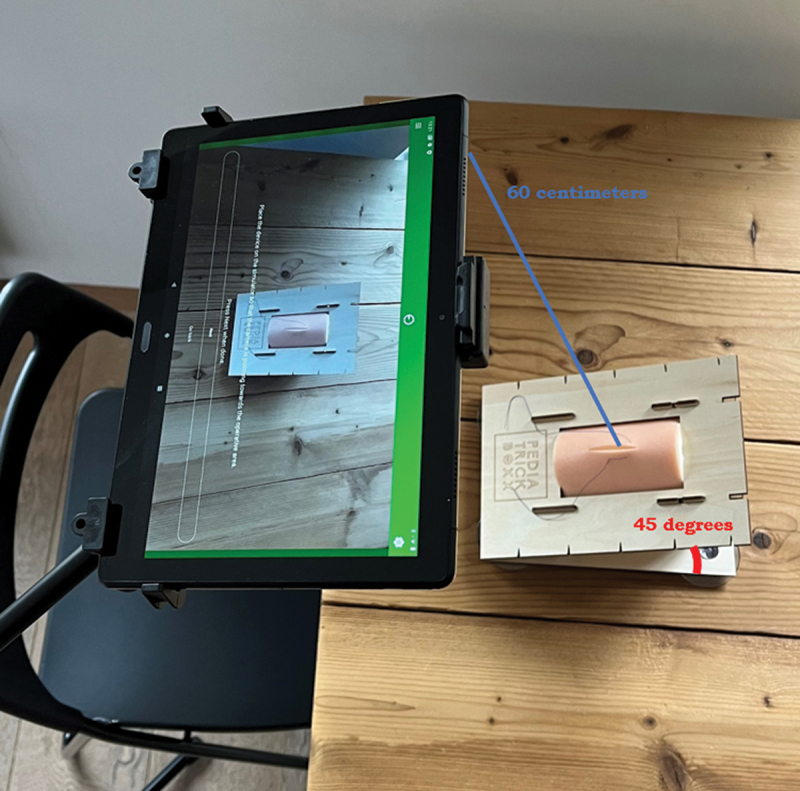
Research setup with a Lenovo P10 tablet in a stand and a simulator by PediatrickBoxx.

For this study, a previously validated simulation set up for the training of basic open suturing skills was used, as shown in Figure 1 [27,28]. The set up consisted of a wooden casing of PediatrickBoxx (Nijmegen, the Netherlands) [29] with a suturing pad from EduStitch (Prinsenbeek, the Netherlands) [30] in it. The suturing pad was placed in the casing, which created a 45-degree angle between the pad and the table, resulting in the possibility to record the tasks with the camera of a tablet. The tablet (Lenovo P10 tablet, Digital River Ireland Ltd) [31] was placed in a stand over the right shoulder of the participant with a distance of 60 cm between the camera and the wooden casing.

SurgTrac software (eoSurgical Ltd., Edinburgh, United Kingdom) [32] was used as a tracking system on the tablet. This software tracked a red and a blue marking, which were placed on the index fingers of the participant over white surgical gloves. The following parameters were gathered by the tracking software: time of executing a task (in seconds), distance traveled by the left and right marking (in meters), distance between hands (average distance between the red and blue marking in centimeters), hands off-screen (in percentage of time), speed (mean, in millimeters/second), acceleration (mean, in millimeters/second^2^), smoothness (mean, in millimeters/second^3^) and handedness (percentage left and right hand usage).

### Tasks

Participants performed four different basic suturing tasks on the simulation set up:

1. ‘Knot tying by hand’ (Figure 2a): participants tied a reef knot consisting of an underhand and overhand throw.

**Figure 2.**
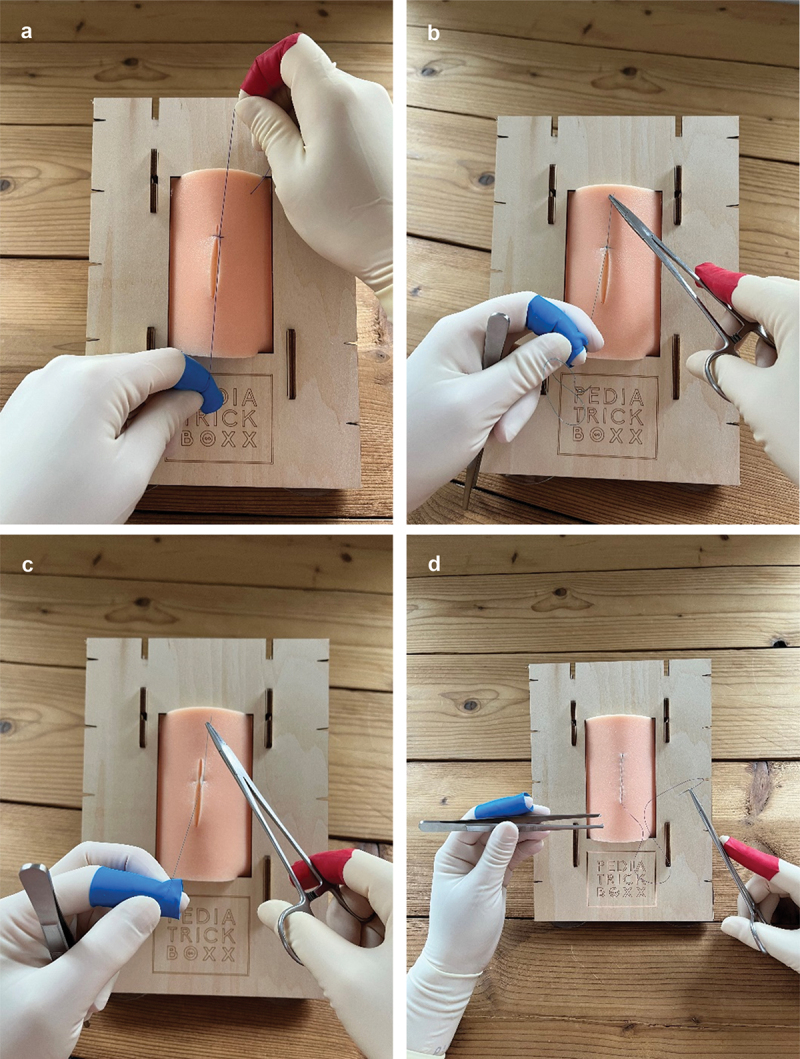
a) Task 1: Knot tying by hand. b) Task 2: Transcutaneous suture. c) Task 3: Vertical mattress suture. d) Task 4: Intracutaneous suture.
2. ‘Transcutaneous suturing’ and knot tying with instruments (Figure 2b): participants executed one transcutaneous suture on the incision of their pad and tied the suture using their instruments.
3. Vertical mattress suturing (‘Donati suture’) and knot tying with instruments (Figure 2c): participants executed one vertical mattress suture on the incision of their pad and tied the suture using their instruments.
4. Continuous ‘intracutaneous suturing’ without knot tying (Figure 2d): an intracutaneous knot was tied in advance by the researcher at the upper side of the incision. Participants performed an intracutaneous suture through the total incision in their pad (4 cm in length). Knot tying was not part of the execution of this task.

### Protocol

All participants completed a form regarding their demographics and suturing experience. Afterwards, they performed the ‘pre-test’, consisting of performing the four different basic suturing tasks while being tracked by the SurgTrac software.

The participants were randomly divided into three groups. The first group (‘clinical exposure group’) consisted of participants who did their surgical rotation at a hospital other than the academic hospital. These participants did not receive additional suturing training and only practiced suturing during the clinical exposure of their surgical rotation.

**Figure 3.**
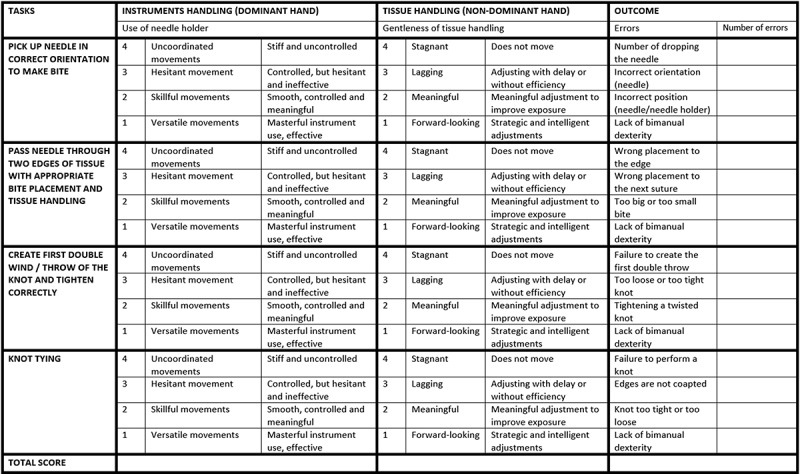
CAT-form with four sub-tasks.

The second group (‘continuous training group’) and third group (‘self-assessment group’) were the participants that did their surgical rotation at the academic hospital (Radboudumc, Nijmegen, The Netherlands). They received a weekly supervised suturing interval training in addition to their clinical exposure. During these sessions, they practiced all four suturing tasks twice, with a maximum duration of 30 min for the total session. In addition to this, the self-assessment group studied a self-assessment form before each training session and before each test to induce and stimulate reflection before practice (Figure 3). The form used, is a Competency (self-)Assessment Tool (CAT), containing four objective subtasks. Each sub-task was assessed for dominant hand/instrument handling, non-dominant hand/tissue handling and step-specific errors. The participants completed this form after each training session and after each test.

**Figure 4.**
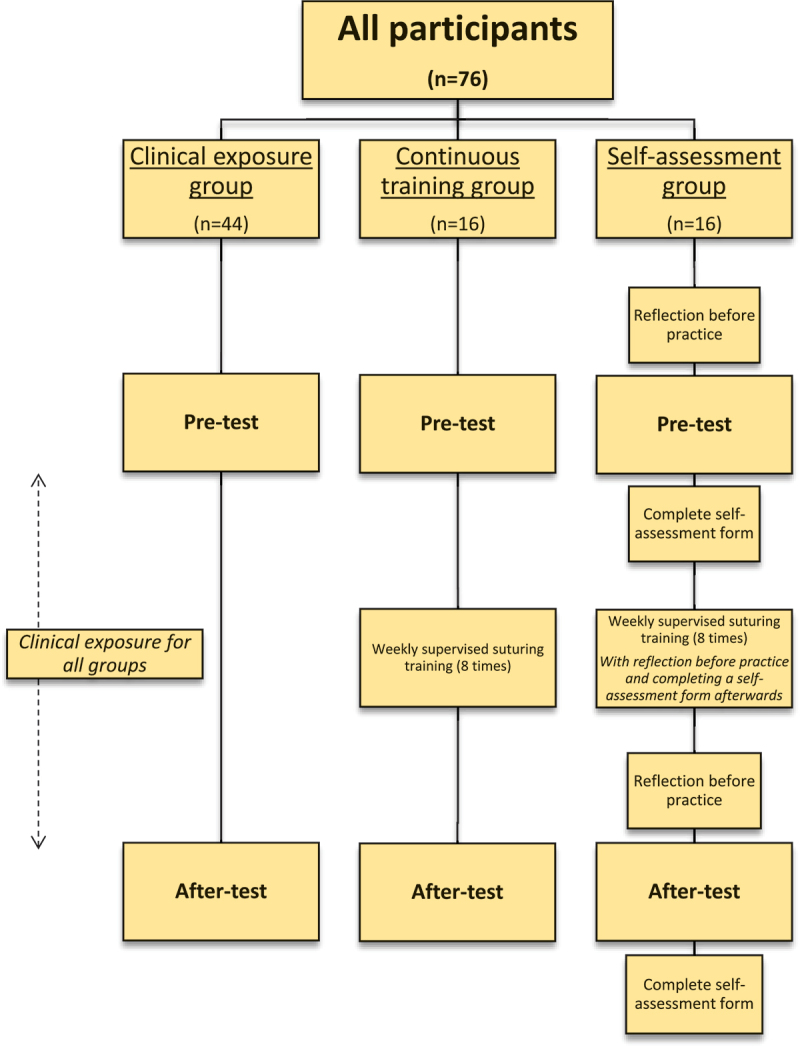
Flowchart of protocol of this study.

At the end of the surgical rotation, the participants of all three groups performed an ‘after-test’. During this test all four suturing tasks were performed again whilst being tracked by the SurgTrac software, as was done during the pre-test. After completion of the test, all participants registered their clinical exposure regarding suturing skills during their surgical rotation. An overview of the protocol is shown in Figure 4.

All participants completed a written informed consent, regarding participation in this study and processing of the data anonymously. A waiver for medical ethical approval was provided, because of the non-medical intervention setup of this study.

### Outcome parameters

The parameters time and distance and a calculated ‘composite score’ served as outcome parameters in this study for the assessment of open suturing tasks. Previous research showed construct validity for the parameters time and distance [33]. Concurrent validity was shown for the parameter time as well as for a calculated composite score [28]. Other measured parameters did not show construct and concurrent validity in previous studies and are therefore not used as outcome parameters

The parameter time was measured as total time needed for the of execution of a task. The parameter distance consisted of the sum of the distance traveled by the red marking and the blue marking. These two parameters were used to calculate a composite score. This composite score is a score on a scale from 0 to 100, with hundred being the optimal score. The formula of this composite score was:

(50 – ((time – time_min_)/time_max_) * 50) + (50 – ((distance – distance_min_)/distance_max_) * 50)

The minimum and maximum of time and distance were determined and validated in the previous research that also included experts [28]. Therefore, if the parameters of the participants of this study exceeded the maximum or minimum ranges of the composite score formula, they would receive the maximal or minimal score for that parameter.

A delta score was defined as the difference between a measured or calculated parameter at the pre-test and the same measured or calculated parameter at the after-test. The formulae of the delta scores were:

Delta score time = (time after-test) – (time pre-test)

Delta score distance = (distance after-test) – (distance pre-test)

Delta score composite score = (composite score after-test) – (composite score pre-test)

### Statistical analyses

Data was analyzed by IBM Statistical Package for Social Sciences (SPSS), version 27. A p-value of <0.05 was considered statistically significant. Due to non-normal distributed data, Wilcoxon S-R tests were used to compare differences between pre-test and after-test in a group. For the differences between groups, Kruskal Wallis tests were used. Afterwards, Mann Whitney U tests were used as post-hoc analysis.

## Results

In total 76 participants were included of whom 44 were in the clinical exposure group, 16 in the continuous training group and 16 in the self-assessment group. The mean age of all participants was 23.7 years (SD 2.7), without significant differences between the groups (*p* = 0.771). Most participants were female (*n* = 57, 75%), this was seen in all three groups.

When comparing the self-reported clinical exposure of the participants during surgical rotation, no significant differences were found between the three groups for Donati suture (*p* = 0.193) and intracutaneous suture (*p* = 0.526). For knot tying by hand, the clinical exposure group had significantly less exposure than the continuous training group (clinical exposure group: 0 ties, continuous training group: 2 ties, *p* = 0.009). The self-assessment group showed no significant differences in execution of knot tying by hand compared to the other two groups. For transcutaneous suture, the clinical exposure group had more clinical exposure than the other two groups (clinical exposure group: 5 sutures, continuous training group: 3 sutures, self-assessment group: 1.5 sutures, *p* = 0.001).

For all tasks, a significant improvement in composite scores was seen for all groups when comparing between the pre-test and the after-test.

### Knot tying by hand (Task 1)

Table 1 shows the outcomes of all groups for Task 1 (knot tying by hand).

**Table 1.** Differences between pre- and after-test in all groups and differences between groups for Task 1 (knot tying by hand). Differences between pre- and after test in a group are calculated with Wilcoxon S-R test. Differences between groups are calculated with Kruskal Wallis tests. *P*-value < 0.05 is considered significant.

Task 1	Test	Clinical exposure (*n* = 44)	Continuous training (*n* = 16)	Self-assessment (*n* = 16)	*p*-value
Time (s) (Median, IQR)	Pre-test	47.5 (50)	34.5 (63)	40.5 (27)	0.919
	After-test	37.0 (45)	23.5 (11)	21.5 (19)	**0.003**
	∆	5.0 (52.3)	11.0 (60.3)	20.0 (15.0)	0.225
	*p*-value	0.121	**0.001**	**0.001**	
Distance (m) (Median, IQR)	Pre-test	3.3 (8.5)	3.9 (9.1)	1.6 (2.1)	0.123
	After-test	1.7 (1.9)	1.6 (1.9)	1.0 (1.5)	0.514
	∆	1.0 (6.9)	1.8 (8.8)	0.4 (1.7)	0.334
	p-value	**0.009**	**0.007**	**0.036**	
Composite score (Median, IQR)	Pre-test	88.0 (15.3)	90.8 (19.4)	92.2 (7.2)	0.602
	After-test	92.9 (9.4)	94.8 (3.1)	96.0 (4.0)	**0.013**
	∆	3.0 (12.9)	3.7 (16.9)	3.7 (2.5)	0.487
	p-value	**0.031**	**0.002**	**<0.001**	

At the pre-test no significant differences were found between groups.

When comparing the three groups at the after-test, the continuous training group and the self-assessment group were significantly faster and had a better composite score than the clinical exposure group (composite score, clinical exposure group: 92.9 vs. continuous training group: 94.8 vs. self-assessment group: 96.0, *p* = 0.013).

### Transcutaneous suture (Task 2)

Table 2 shows the outcomes of all groups for Task 2 (transcutaneous suture).

**Table 2.** Differences between pre- and after-test in all groups and differences between groups for Task 2 (transcutaneous suture). Differences between pre- and aftertest in a group are calculated with Wilcoxon S-R test. Differences between groups are calculated with Kruskal Wallis tests. *P*-value < 0.05 is considered significant.

Task 2	Test	Clinical exposure (*n* = 44)	Continuous training (*n* = 16)	Self-assessment (*n* = 16)	*p*-value
Time (s) (Median, IQR)	Pre-test	101.5 (46)	119.5 (61)	95.0 (45)	0.080
	After-test	53.5 (32)	46.0 (22)	50.0 (37)	0.458
	∆	55.5 (36)	69.5 (49.3)	37.0 (28)	**0.013**
	p-value	**<0.001**	**<0.001**	**<0.001**	
Distance (m) (Median, IQR)	Pre-test	8.1 (10.0)	8.7 (12.5)	5.0 (6.7)	0.235
	After-test	3.1 (4.3)	2.9 (3.2)	4.0 (2.6)	0.741
	∆	3.9 (8.5)	5.4 (15.6)	2.4 (4.2)	0.234
	p-value	**<0.001**	**0.017**	**0.008**	
Composite score (Median, IQR)	Pre-test	67.9 (15.9)	62.3 (21.9)	74.1 (19.6)	0.063
	After-test	89.3 (12.9)	87.7 (9.8)	86.7 (8.7)	0.733
	∆	18.8 (17.6)	25.8 (29.6)	13.7 (13.6)	0.061
	p-value	**<0.001**	**<0.001**	**<0.001**	

No significant differences were found between groups at the pre-test. However, when comparing time between groups, the self-assessment group was faster compared to the clinical exposure group and continuous training group (clinical exposure group: 101.5 vs. continuous training group: 119.5 vs. self-assessment group: 95.0, *p* = 0.080), resulting in a significant smaller delta between time at both test moments (clinical exposure group: 55.5 vs. continuous training group: 69.5 vs. self-assessment group: 37.0, *p* = 0.013).

At the after-test, no significant differences were found between groups (composite score: 89.3 vs. 87.7 vs. 86.7, *p* = 0.733).

### Donati suture (Task 3)

Table 3 shows the outcomes of all groups for Task 3 (Donati suture).

**Table 3.** Differences between pre- and after-test in all groups and differences between groups for Task 3 (Donati suture). Differences between pre- and aftertest in a group are calculated with Wilcoxon S-R test. Differences between groups are calculated with Kruskal Wallis tests. *P*-value <0.05 is considered significant.

Task 3	Test	Clinical exposure (*n* = 44)	Continuous training (*n* = 16)	Self-assessment (*n* = 16)	*p*-value
Time (s) (Median, IQR)	Pre-test	184.0 (87)	171.5 (64)	151.0 (44)	0.068
	After-test	103.5 (46)	87.5 (40)	90.5 (47)	0.348
	∆	69.5 (57.8)	75.5 (60.8)	56.0 (51.5)	0.228
	p-value	**<0.001**	**<0.001**	**<0.001**	
Distance (m) (Median, IQR)	Pre-test	16.9 (19.1)	13.8 (14.8)	7.5 (10.3)	**0.004**
	After-test	5.2 (6.3)	4.8 (7.8)	6.7 (5.0)	0.670
	∆	8.7 (18.2)	8.1 (16.3)	1.6 (8.3)	**0.005**
	p-value	**<0.001**	**0.015**	0.301	
Composite score (Median, IQR)	Pre-test	64.2 (27.7)	64.2 (14.7)	75.3 (12.1)	**0.004**
	After-test	83.6 (8.5)	85.4 (10.8)	83.5 (10.9)	0.818
	∆	22.7 (23.3)	16.4 (14.8)	10.1 (7.1)	**0.007**
	p-value	**<0.001**	**<0.001**	**0.001**	

When comparing the three groups at the pre-test, a significant better composite score was found for the self-assessment group (clinical exposure group: 64.2 vs. continuous training group: 64.2 vs. self-assessment group: 75.3, *p* = 0.004). Furthermore, the self-assessment group scored a significant smaller delta in composite score than the other two groups (clinical exposure group: 22.7 vs. continuous training group: 16.4 vs. self-assessment group: 10.1, *p* = 0.007).

At the after-test no significant differences were found between the three groups (composite score: 83.6 vs. 85.4 vs. 83.5, *p* = 0.818).

### Intracutaneous suture (Task 4)

Table 4 shows the outcomes of all groups for Task 4 (intracutaneous suture).

**Table 4.** Differences between pre- and after-test in all groups and differences between groups for Task 4 (intracutaneous suture). Differences between pre- and aftertest in a group are calculated with Wilcoxon S-R test. Differences between groups are calculated with Kruskal Wallis tests. P-value <0.05 is considered significant.

Task 4	Test	Clinical exposure (*n* = 44)	Continuous training (*n* = 16)	Self-assessment (*n* = 16)	p-value
Time (s) (Median, IQR)	Pre-test	310.5 (128)	281.0 (102)	292.0 (109)	0.823
	After-test	223.5 (136)	199.0 (114)	247.0 (35)	0.356
	∆	76.5 (151.5)	87.5 (83.3)	58.5 (117.5)	0.622
	p-value	**<0.001**	**0.002**	**0.016**	
Distance (m) (Median, IQR)	Pre-test	29.9 (29.0)	24.3 (24.6)	10.5 (13.0)	**<0.001**
	After-test	14.4 (20.3)	12.3 (10.0)	12.6 (12.3)	0.689
	∆	13.3 (29.0)	9.5 (16.0)	−1.2 (11.4)	**0.009**
	p-value	**<0.001**	**0.015**	0.918	
Composite score (Median, IQR)	Pre-test	59.4 (32)	64.4 (21.1)	70.6 (17.4)	0.063
	After-test	74.8 (19.3)	79.7 (12.0)	73.9 (8.2)	0.495
	∆	17.4 (27.9)	14.8 (15.87)	5.4 (16.3)	0.055
	p-value	**<0.001**	**0.003**	**0.049**	

At the pre-test the self-assessment group scored better compared to the other two groups with a higher composite score (clinical exposure group: 59.4 vs. continuous training group: 64.4 vs. self-assessment group: 70.6, *p* = 0.063), resulting in a smaller delta between the composite scores at the two test moments in this group (clinical exposure group: 17.4 vs. continuous training group: 14.8 vs. self-assessment group: 5.4, *p* = 0.055).

No significant differences were found when comparing the composite scores between groups at the after-test (composite score: 74.8 vs. 79.7 vs. 73.9, *p* = 0.495).

## Discussion

This study indicates that reflection before practice on basic open suturing skills may improve surgical skills at the start of the learning curve and thus may shorten the learning process for these tasks. The self-assessment group studied a self-assessment form before starting the pre-test resulting in reflection before practice. They completed the tasks in a shorter amount of time, with a shorter distance and/or had a higher composite score than the other groups for transcutaneous, Donati and intracutaneous suturing at the pre-test. This suggests that reflection before practice by studying a self-assessment form results in a higher skills-level at the start of the learning curve.

Participants were randomly assigned to the different groups and the self-assessment group received and studied their self-assessment form before they start the test and therefore knew on which assessment-parameters they had to focus. This could explain the higher objective scores of the self-assessment group at the start of their learning curve. It emphasises that reflection before practice has a positive effect on performance, which is in line with literature about other skills [24–26].

Even though two out of three groups (continuous training group and self-assessment group) had weekly supervised suturing training on top of their clinical exposure, no significant differences in composite score at the after-test were found for transcutaneous, Donati and intracutaneous suturing for these groups, when compared to the clinical exposure group. This suggests that continuous training alone, when done in addition to clinical exposure, was not responsible for the observed positive effect.

For knot tying by hand the continuous training group and self-assessment group both scored better at the after-test compared to the clinical exposure group. This could be caused by either the continuous training that first mentioned groups received or the difference in clinical exposure during the surgical rotation. The clinical exposure group reported less exposure to this task during rotation than the continuous training group; however, their exposure was similar to that of the self-assessment group. Nevertheless, the self-assessment group scored best on this task, suggesting that reflection before practice may play an important role.

Although all three groups of medical students improved their basic suturing skills during surgical rotation; the self-assessment group had smaller difference in score when comparing the pre-test and after-test, suggesting a shorter learning curve for this group. At the after-test no significant differences were found between the groups. Further research could focus on the skills level after a few months, to evaluate whether the training schedule and self-assessment affects skill retention over time.

Clinical exposure to open surgical skills decreased over the last few years [1–3]. Several reasons have been mentioned, including shorter training programs for surgeons, the COVID-19 pandemic and the focus on laparoscopic and robotic techniques [1,3,34]. This has led to temporary reorganisation of health care and adjustment of several surgical programs, eventually leading to less exposure in the OR [1–3,35,36]. Nevertheless, in the context of patient safety, medical students, surgical residents and junior staff members are expected to acquire and maintain surgical skills. This indicates the importance of surgical simulation training. In the previous research, a simulation set up for open surgical training was developed [27], and construct and concurrent validity were established [28]. However, an adequate training schedule to use this simulation set up was lacking.

In this study continuous training using reflection before practice and self-assessment and seemed to be the most promising addition to clinical exposure during a surgical rotation, compared to clinical exposure only or continuous training without reflection before practice and self-assessment. With reflection before practice, medical students start their surgical rotation at a higher skills level, which can compensate the diminishing clinical exposure in a later stage of their curriculum. Future research could explore whether reflection before practice and self-assessment during a surgical rotation without continuous training has the same effect on the learning curve as reflection before practice and self-assessment with continuous suturing training.

### Limitations

In this study, the self-assessment group used reflection before practice before all tests, including the pre-test. The baseline skills level of this group was not measured. All participants were randomly divided in groups and thus no difference in baseline skills level between groups was expected. However, bias in baseline skills level could not be completely excluded.

A significant difference in clinical exposure was found between groups for transcutaneous suture. The clinical exposure group reported significantly more exposure to transcutaneous sutures compared to both continuous training group and self-assessment group. Analysis showed that continuous training had no positive effect when this is done in addition to clinical exposure during a surgical rotation for transcutaneous suturing. For this task, however, the effect could be disguised by the differences in clinical exposure between groups during surgical rotation.

Another limitation of the study is that it took place at different (teaching) hospitals. Despite the fact that in The Netherlands the surgical rotation program is standardized, there are obviously differences in different clinical hospital settings. However, randomizing participants to the different groups should have reduced a considerable part of this bias.

This study is performed during COVID-19. This could have affected the amount of clinical exposure. It could be interesting to evaluate the clinical exposure after COVID-19 and analyse if there is still no difference in clinical exposure for medical students in an academic and non-academic hospital.

## Conclusion

This study showed that reflection before practice and self-assessment during training and tests of basic open surgical skills, resulted in a higher starting level at the beginning of the learning curve and could therefore be of added value. However, continuous simulation training of basic open suturing skills without reflection before practice and self-assessment as an addition to clinical exposure to suturing did not result in higher skill levels as tested at the end of the surgical rotation.

## Data Availability

The datasets generated and analyzed during the current study are available from the corresponding author on reasonable request.

## References

[1] Juprasert JM, Gray KD, Moore MD, et al. Restructuring of a general surgery residency program in an epicenter of the coronavirus disease 2019 pandemic: lessons from New York City. JAMA Surg. 2020;155(9):870–10. doi: 10.1001/jamasurg.2020.3107 32936281

[2] Jamal MH, Wong S, Whalen TV. Effects of the reduction of surgical residents’ work hours and implications for surgical residency programs: a narrative review. BMC Med Educ. 2014;14 Suppl 1(Suppl 1):S14. doi: 10.1186/1472-6920-14-S1-S14 25560685 PMC4304271

[3] Munro C, Burke J, Allum W, et al. Covid-19 leaves surgical training in crisis. BMJ. 2021;372:n659. doi: 10.1136/bmj.n659 33712499

[4] Scerbo MW, Britt RC, Montano M, et al. Effects of a retention interval and refresher session on intracorporeal suturing and knot tying skill and mental workload. Surgery. 2017;161(5):1209–1214. doi: 10.1016/j.surg.2016.11.011 28011014 PMC5404977

[5] Bekele A, Wondimu S, Firdu N, et al. Trends in retention and decay of basic surgical skills: evidence from Addis Ababa University, Ethiopia: a prospective case-control cohort study. World J Surg. 2019;43(1):9–15. doi: 10.1007/s00268-018-4752-1 30097707

[6] Varley M, Choi R, Kuan K, et al. Prospective randomized assessment of acquisition and retention of SILS skills after simulation training. Surg Endosc. 2015;29(1):113–118. doi: 10.1007/s00464-014-3647-y 25005011

[7] Weis JJ, Farr D, Abdelfattah KR, et al. A proficiency-based surgical boot camp May not provide trainees with a durable foundation in fundamental surgical skills. Am J Surg. 2019;217(2):244–249. doi: 10.1016/j.amjsurg.2018.07.040 30057109

[8] Kesser BW, Hallman M, Murphy L, et al. Interval vs massed training: how best do we teach surgery? Otolaryngol Head Neck Surg. 2014;150(1):61–67. doi: 10.1177/0194599813513712 24270165

[9] Van Bruwaene S, Schijven MP, Miserez M. Maintenance training for laparoscopic suturing: the quest for the perfect timing and training model: a randomized trial. Surg Endosc. 2013;27(10):3823–3829. doi: 10.1007/s00464-013-2981-9 23660721

[10] Joosten M, Bökkerink GMJ, Stals JJM, et al. The effect of an interval training on skill retention of high-complex low-volume minimal invasive pediatric surgery skills: a pilot study. J Laparoendosc Adv Surg Tech A. 2021;31(7):820–828. doi: 10.1089/lap.2020.1024 33944585

[11] Schoeff S, Hernandez B, Robinson DJ, et al. Microvascular anastomosis simulation using a chicken thigh model: Interval versus massed training. Laryngoscope. 2017;127(11):2490–2494. doi: 10.1002/lary.26586 28407264

[12] Spruit EN, Band GPH, van der Heijden KD, et al. The effects of spacing, naps, and fatigue on the acquisition and retention of laparoscopic skills. J Surg Educ. 2017;74(3):530–538. doi: 10.1016/j.jsurg.2016.11.003 27988169

[13] Cecilio-Fernandes D, Cnossen F, Jaarsma D, et al. Avoiding surgical skill decay: a systematic review on the spacing of training sessions. J Surg Educ. 2018;75(2):471–480. doi: 10.1016/j.jsurg.2017.08.002 28843958

[14] Joosten M, Bökkerink GMJ, Verhoeven BH, et al. Are self-assessment and peer assessment of added value in training complex pediatric surgical skills? Eur J Pediatr Surg. 2020;31(1):025–033. doi: 10.1055/s-0040-1715438 32772347

[15] Nayar SK, Musto L, Baruah G, et al. Self-assessment of surgical skills: a systematic review. J Surg Educ. 2020;77(2):348–361. doi: 10.1016/j.jsurg.2019.09.016 31582350

[16] Schock S, Shaver SL, Craigen B, et al. Educational research report correlation between student self-assessment and proctor evaluation in a veterinary surgical laboratory. J Vet Med Educ. 2021;48(5):584–591. doi: 10.3138/jvme-2019-0096 33226902

[17] Ganni S, Chmarra MK, Goossens RHM, et al. Self-assessment in laparoscopic surgical skills training: is it reliable? Surg Endosc. 2017;31(6):2451–2456. doi: 10.1007/s00464-016-5246-6 27655377 PMC5443853

[18] Tobias KM, Bailey MR. Veterinary student self-assessment of basic surgical skills as an experiential learning tool. J Vet Med Educ. 2020;47(6):661–667. doi: 10.3138/jvme.2018-0004 32053054

[19] Netter A, Schmitt A, Agostini A, et al. Video-based self-assessment enhances laparoscopic skills on a virtual reality simulator: a randomized controlled trial. Surg Endosc. 2021;35(12):6679–6686. doi: 10.1007/s00464-020-08170-7 33241429

[20] Thinggaard E, Zetner DB, Fabrin A, et al. A study of surgical residents’ self-assessment of open surgery skills using gap analysis. Simul Healthc. 2023;18(5):305–311. doi: 10.1097/SIH.0000000000000694 36730862

[21] Balvardi S, Kaneva P, Semsar-Kazerooni K, et al. Effect of video-based self-reflection on intraoperative skills: a pilot randomized controlled trial. Surgery. 2024;175(4):1021–1028. doi: 10.1016/j.surg.2023.11.028 38154996

[22] Alam L, Alam M, Shafi MN, et al. Meaningful in-training and end-of-training assessment: the need for implementing a continuous workplace-based formative assessment system in our training programs. Pak J Med Sci. 2022;38(5):1132–1137. doi: 10.12669/pjms.38.5.5921 35799747 PMC9247791

[23] Mackenzie H, Cuming T, Miskovic D, et al. Design, delivery, and validation of a trainer curriculum for the national laparoscopic colorectal training program in England. Ann Surg. 2015;261(1):149–156. doi: 10.1097/SLA.0000000000000437 24374538

[24] Ganni S, Botden S, Schaap DP, et al. “Reflection-before-practice” improves self-assessment and end-performance in laparoscopic surgical skills training. J Surg Educ. 2018;75(2):527–533. doi: 10.1016/j.jsurg.2017.07.030 28822819

[25] Edwards S. Reflecting differently. New dimensions: reflection-before-action and reflection-beyond-action. Int Pract Devel J. 2017;7(1):1–14. doi: 10.19043/ipdj.71.002

[26] Botelho M, Bhuyan SY. Reflection before and after clinical practice—enhancing and broadening experience through self‐, peer‐and teacher‐guided learning. Eur J Dent Educ. 2021;25(3):480–487. doi: 10.1111/eje.12623 33190406

[27] Hillemans V, Verhoeven B, Botden S. Feasibility of tracking in open surgical simulation. Int J Healthcare Simul. 2022;1–10. doi: 10.54531/juvj5939

[28] Hillemans V, van de Mortel X, Buyne O, et al. Objective assessment for open surgical suturing training by finger tracking can discriminate novices from experts. Med Educ Online. 2023;28(1):2198818. doi: 10.1080/10872981.2023.2198818 37013910 PMC10075519

[29] Bökkerink GM. PediatrickBoxx Available from: https://www.pediatrickboxx.com/

[30] EduStitch. Available from: https://www.edustitch.com/

[31] Lenovo. Available from: https://www.lenovo.com/nl/nl/tablets/android-tablets/lenovo-tab-series/Tab-P10/p/ZZITZTATB7X?orgRef=https%253A%252F%252Fwww.google.com%252F

[32] Ferns J. An app to make a surgeon. BMJ. 2013;346:f3361. doi: 10.1136/sbmj.f3361

[33] Hillemans V, van de Mortel X, Buyne O, et al. Objective assessment for open surgical suturing training by finger tracking can discriminate novices from experts. Med Educ Online. 2022;28(1). doi: 10.1080/10872981.2023.2198818 PMC10075519 37013910

[34] Sheetz KH, Claflin J, Dimick JB. Trends in the adoption of robotic surgery for common surgical procedures. JAMA Netw Open. 2020;3(1):e1918911. doi: 10.1001/jamanetworkopen.2019.18911 31922557 PMC6991252

[35] Wendt S, Abdullah Z, Barrett S, et al. A virtual COVID-19 ophthalmology rotation. Surv Ophthalmol. 2021;66(2):354–361. doi: 10.1016/j.survophthal.2020.10.001 33058927 PMC7550053

[36] St John A, Caturegli I, Kubicki NS, et al. The rise of minimally invasive surgery: 16 year analysis of the progressive replacement of open surgery with laparoscopy. JSLS. 2020;24(4):e2020.00076. doi: 10.4293/JSLS.2020.00076 PMC7810432 33510568

